# Corilagin ameliorates the extreme inflammatory status in sepsis through TLR4 signaling pathways

**DOI:** 10.1186/s12906-016-1533-y

**Published:** 2017-01-05

**Authors:** Hua-Rong Li, Jie Liu, Shu-Ling Zhang, Tao Luo, Fei Wu, Ji-Hua Dong, Yuan-Jin Guo, Lei Zhao

**Affiliations:** 1 Department of Infectious Diseases, Union Hospital, Tongji Medical College, Huazhong University of Science and Technology, Wuhan, 430022 People’s Republic of China; 2 Department of Integrated Traditional Chinese and Western Medicine, Union Hospital, Tongji Medical College, Huazhong University of Science and Technology, Wuhan, 430022 People’s Republic of China; 3 Department of Intensive Care Unit, Renmin Hospital, Hubei University of Medicine, Shiyan, 442000 People’s Republic of China; 4 Department of Neurology, Union Hospital, Tongji Medical College, Huazhong University of Science and Technology, Wuhan, 430022 People’s Republic of China; 5 Department of Neurology, Wuhan General Hospital of Guangzhou Military Command, Wuhan, 430070 People’s Republic of China; 6 Central Lab, Union Hospital, Tongji Medical College, Huazhong University of Science and Technology, Wuhan, 430022 People’s Republic of China

**Keywords:** Corilagin, Epsis, TLR4, Signal pathway, Pro-inflammatory cytokines

## Abstract

**Background:**

Sepsis is one of the serious disorders in clinical practice. Recent studies found toll-like receptors 4 (TLR4) played an important role in sepsis. In this study, we tried to find the influence of Corilagin on TLR4 signal pathways *in vitro* and *in vivo*.

**Methods:**

The cellular and animal models of sepsis were established by LPS and then interfered with Corilagin. Real-time PCR and western blot were employed to detect the mRNA and protein expressions of TLR4, MyD88, TRIF and TRAF6. ELISA was used to determine the IL-6 and IL-1β levels in supernatant and serum.

**Results:**

The survival rate was improved in the LPS + Corilagin group, and the mRNA and protein expressions of TLR4, MyD88, TRIF and TRAF6 were significantly decreased than that in the LPS group both in cellular and animal models (*P* < 0.01). The pro-inflammatory cytokines IL-6 and IL-1β were greatly decreased in the LPS + Corilagin group both in supernatant and serum (*P* < 0.01).

**Conclusions:**

Corilagin exerts the anti-inflammatory effects by down-regulating the TLR4 signaling molecules to ameliorate the extreme inflammatory status in sepsis.

## Background

Sepsis is one of the serious complications of trauma, shock, burns and other critical patients, and can induce multiple organ dysfunction syndromes (MODS) [1]. The core mechanism of sepsis is inflammation. During the process of serious infection, pro-inflammatory cytokines pour out, leading to systemic inflammation reaction syndromes (SIRS) [2]. Sepsis and SIRS are the different phases of same pathological process.

Recently, studies found toll-like receptors (TLRs) played an important role in sepsis, especially in early stage as producing of pro-inflammatory cytokines [3]. Recognition of pathogens and persistent systemic inflammation cascade are linked by TLRs. Thereby, regulation of TLRs may ameliorates conditions of progression and prognosis of sepsis [4, 5]. TLR4 is the first toll-like associated protein found in the human and is distributed in B/T lymphocytes, macrophages, liver, spleen and lung [6]. Gram-negative bacterial LPS is the mainly ligand. As gram-negative bacteria are the main pathogens in sepsis, in critical patients TLR4 as the main protein in natural immunity plays a vital role in activating monocyte-macrophage system, triggering inflammatory cascade, intensifying inflammation, and resulting in sepsis eventually [7, 8].

TLR4 signals were transduced through MyD88 and TRIF pathways. The classic MyD88 pathway plays a key role in production of pro-inflammatory cytokines. TLR4 receives stimulation such as LPS, sharing downstream molecule TIRAP with TLR2. Then MyD88 is activated and TRAF6 receives the signal and promotes the production of pro-inflammatory cytokines. Additionally, MAPK and NEMO take part in the generation as well [9]. Meanwhile, in TRIF pathway TLR4 sharing downstream molecule TRAM with TLR3, and then TRAF6 receives signals to produce pro-inflammatory cytokines. Simultaneously, activated TRAF3 promotes the type I interferon via IRF3. Thereby, focusing on TLR4 and key molecules in the signaling pathways may help to find a new way to understand controlling extreme inflammatory status in sepsis.

Corilagin (C_27_H_22_O_18_) is identified in several plants [10]. Our previous studies has found that Corilagin was effective in anti-inflammation, hepatic protection, anti-oxidant and anti-viral encephalitis [11–13]. Corilagin could also ameliorate schistosomiasis-associated liver fibrosis by in S. japonicum-infected Balb/c mice and IL-13-induced macrophages and hepatic satellite cells [14–17]. Based on anti-inflammatory and antibacterial activity of Corilagin and our experience on inflammation [18], we would explore the effect of Corilagin to treat sepsis by interfering with TLR4 signaling pathway.

In this study, we set up the cellular model with RAW264.7 cells stimulated by LPS and established sepsis animal model and then administered the models with Corilagin, hoping to find the exact mechanisms of Corilagin in ameliorating sepsis by interfering with TLR4-associated pathways.

## Methods

### Chemicals and reagents

Trizol reagent, RNAios and Real-time PCR kit were obtained from TaKaRa Company (Dalian, China). Anti- MyD88, anti-TRAF6, anti-TRIF and anti-TLR4 antibodies were offered from Abcam Biotechnology (MA, USA). LPS was taken from Sigma-Aldrich (MO, USA). The immunohistochemistry kits and HRP-labeled secondary antibodies were bought from Boster Biotechnology (Wuhan, China). The IL-1β and IL-6 ELISA kit were purchased from Dkewei Biotechnology (Beijing, China). Corilagin was received from China National institutes for food and drug control.

### Cell administration

RAW264.7 cells were bought from Xiangya Central Laboratory. The cells were cultured in DMEM medium containing 10% fetal bovine serum (FBS) in the incubator at 37 °C, 5% CO_2_ and saturated humidity. As the cells grew up to 80%, serum-free DMEM medium was added for incubation overnight. RAW264.7 cells were divided into three groups, the Corilagin group, model group and normal group. The Corilagin group was stimulated by LPS (1 mg/ml) for 2 h and then Corilagin (0.25 mg/ml) was added. The model group incubated with LPS (1 mg/ml) only. The normal group without any treatment was as negative control. 24 h later the cells were harvested for total RNA isolation and proteins extraction. The cultural supernatants were collected for ELISA. The procedures were repeated for three times.

### Animals and treatment

Thirty male Balb/c mice, weighting 19 ~ 25 g, 6 ~ 8 weeks old, were purchased from Hubei Provincial Center for Disease and Prevention. The mice were feed in 22 ~ 25 °C temperature and 50-60% humidity for one week. Then the animals were randomly divided into 3 groups, the LPS + Corilagin group, LPS group and control group, with 10 mice in each group. The LPS + Corilagin group and LPS group were given LPS 20 mg/kg intraperitoneally. The LPS + Corilagin group was given Corilagin (40 mg/kg) orally after LPS administration, and the LPS group was administrated with equivalent volume of saline. The control group was given normal saline intraperitoneally and orally instead of LPS and Corilagin. 12 h later, all the mice were sacrificed after anesthetized by the 10% chloral hydrate. The liver tissues were stored in a refrigerator of minus −80 °C for total RNA extraction and western-blot testing. All animal study protocols were approved by the Ethics Committee of Huazhong University of Science and Technology [19].

### Histological examination

The liver, colon, lung, brain and kidney tissues were fixed in 4% formalin and embedded in paraffin. Four micrometers serial sections were obtained for haematoxylin and eosin (HE) staining to observe the liver pathological changes. The procedure was referenced by our past experiment [20, 21].

### Total RNA isolation and quantitative real-time PCR

The procedure abided by our past experiment [22]. Total RNA was isolated from the liver tissues by using RNAiso Plus (Takara, Dalian, China), and reverse transcription was performed following the protocol of kit (TaKaRa Primescript RT Master Mix Perfect Real Time. Code: DRR036S). Then the samples were incubated at 37 °C for 15 min and at 85 °C for 5 s. After the template cDNA was synthesized the amplification steps were followed with the instruction of kit (TaKaRa SYBR Premix Ex Taq, Code: DRR041S) and the reaction conditions were 95 °C 30 s; 95 °C 5 s, 60 °C 30 s, 40 cycles; 95 °C 15 s, 60 °C 1 min, 95 °C 15 s. The results were analyzed by using 2-△△CT method. GAPDH was used as the internal control. The procedure were repeated for three times. All the primers for quantitative real-time PCR included as follows.

GAPDH-forward,5’-TGTGTCCGTCGTGGATCTGA-3’,

GAPDH-reverse, 5’-CCTGCTTCACCACCTTCTTGA-3’,

TLR4-forward, 5’-CTCTGGGGAGGCACATCTT -3’

TLR4-reverse, 5’-CTGCTGTTTGCTCAGGATTC -3’

MyD88-forward, 5’- GCCAGAGTGGAAAGCAGTGT -3’

MyD88- reverse, 5’- CGTTGGGGCAGTAGCAGATA -3’

TRIF-forward, 5’- GCAGAGTCGGGGTAACAAGA -3’

TRIF-reverse, 5’- CCAGAAGGTGGTGCTCAAATA -3’

TRAF6-forward, 5’- GCCGAAATGGAAGCACAG -3’

TRAF6-reverse, 5’- CAGGGCTATGGATGACAACA -3’

### Western blot analysis

The procedure was followed by our past experiment [23, 24]. The expression of TLR-4 and associated signaling molecule proteins were detected by western-blot assay. The cells of liver tissue proteins (50 μg) from each sample were separated on SDS-PAGE, and then transferred to nitrocellulose filter membranes which were blocked overnight with 5% nonfat milk in TBST. Blots were probed over night at 4 °C with rabbit polyclonal TLR4 (diluted as 1:500), MyD88 (diluted as 1:500), TRIF (diluted as 1:500) and TRAF6 (diluted as 1:500) and then were followed by incubated with HRP-labeled secondary antibody for two hours before being washed 5–6 times in TBST. After further washing with TBST, ECL was added to identify the immunoreactive bands. The densitometry analysis of the immunoreactive bands was performed using the Fuji ultrasonic-doppler velocity profile (UVP) system and Image J program. The procedure was repeated for three times.

### Detection of IL-1β and IL-6 expression by ELISA

The procedure abided by our past experiment [25]. The expressions of IL-1β and IL-6 in cell supernatants and mice serum were tested by ELISA. Prepare all reagents before starting assay procedure. All standards and samples were added in 5 duplicates. For setting standard wells, the standard was diluted to 1000 pg/ml, 500 pg/ml, 250 pg/ml, 125 pg/ml, 62.5 pg/ml, 31.25 pg/ml, 15.625 pg/ml, 0 pg/ml in standard wells. Then the 100ul samples were added to testing well and 50ul of biotinylated antibody (1:100) were added to each well. By covering with an adhesive strip and the plate was incubated for 90 min at 37 °C. Then each well was washed for 4 times, and each well was added with 100ul diluted streptavidin-HRP (1:100) and incubated for 30 min at 37 °C. After repeating the washing process, 100ul TMB was added to each well and the plate was incubated for 10 min at 37 °C before the stop solution was added. The standard curve was generated by plotting the average 50 nm OD.

### Statistical analysis

The statistics were expressed as the means ± SE. Comparisons between groups were performed with one-way ANOVA test and Student’s *t*-test. All data were analyzed by using SPSS12.0 statistic software. *P* < 0.05 was regard as statistically significant difference [26].

## Results

Effect of Corilagin on mRNA expression of TLR4, MyD88, TRIF and TRAF6 in cellular model

**Fig. 1:**
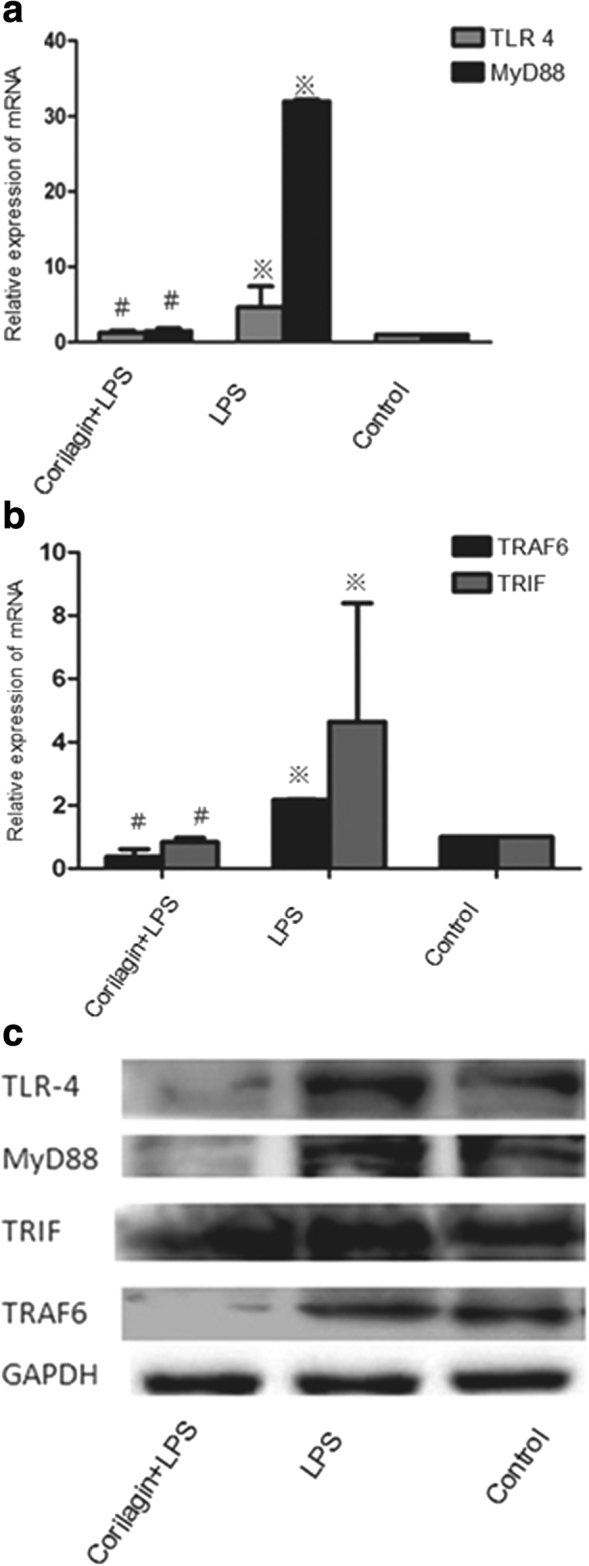
The mRNA and protein expression of TLR4, MyD88, TRAF6 and TRIF in cellular models. **a** Effect of Corilagin on mRNA expression of TLR4 and MyD88 in cellular model. **b** Effect of Corilagin on mRNA expression of TRAF6 and TRIF in cellular model.※p < 0.01 compared with the control group. #p < 0.01 compared with the LPS group. **c** Effect of corilagin on TLR4, MyD88, TRIF and TRAF6 protein expression in cellular model. Western blot assay showed the TLR4, MyD88, TRIF and TRAF6 proteins expression were greatly increased in LPS group, while in the Corilagin + LPS groups, the proteins were decreased compared with that in the LPS group

In the LPS group the mRNA levels of TLR4, MyD88, TRIF and TRAF6 were significantly increased when compared with the control group (*p* < 0.01). With Corilagin intervention, the gene expression of TLR4, MyD88, TRIF and TRAF6 were significantly reduced when compared with Corilagin + LPS group (*p* < 0.01) (Fig. 1a and b).

### Effect of Corilagin on protein expression of TLR4, MyD88, TRIF and TRAF6 in cellular model

Western-blotting assay was employed to test the changes of TLR4, MyD88, TRIF and TRAF6 proteins (Fig. 1c). The proteins were greatly increased in LPS group when compared with the control group, while in Corilagin + LPS groups the proteins were decreased when compared with the LPS group.

### Effect of Corilagin on expression of IL-1β, IL-6 in cell supernatants

**Fig. 2:**
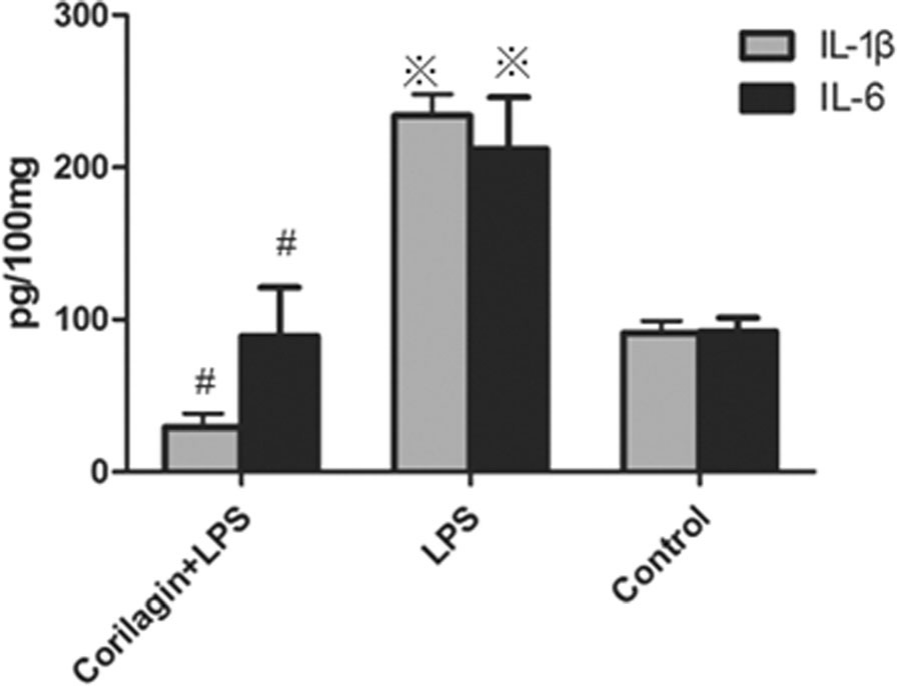
Effect of Corilagin on expression of IL-1β, IL-6 in cell supernatants. Effect of Corilagin on expression of IL-1β, IL-6 in cell supernatants. ※p < 0.01 compared with the control group. #p < 0.01 compared with the LPS group

The expression of IL-1β and IL-6 in cell supernatants was tested by ELISA. In the LPS group the IL-1β, IL-6 levels were significantly increased when compared with the control group (*p* < 0.01). With Corilagin intervention, IL-1β, IL-6 expression were significantly reduced when compared with the LPS group (*p* < 0.01) (Fig. 2).

### Effect of Corilagin on survival rate in animal model

**Fig. 3:**
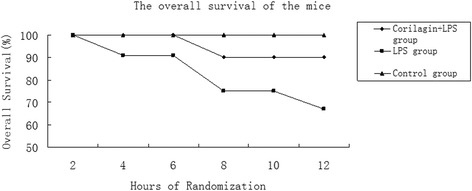
The overall survival of the mice. Within 12 h, mice survival in Corilagin + LPS group was much higher than in LPS group

Mice survival rate in LPS + Corilagin group was much higher than that in LPS group. (Fig. 3).

Effect of Corilagin on mRNA expression of TLR4, MyD88, TRIF and TRAF6 in animal model

**Fig. 4:**
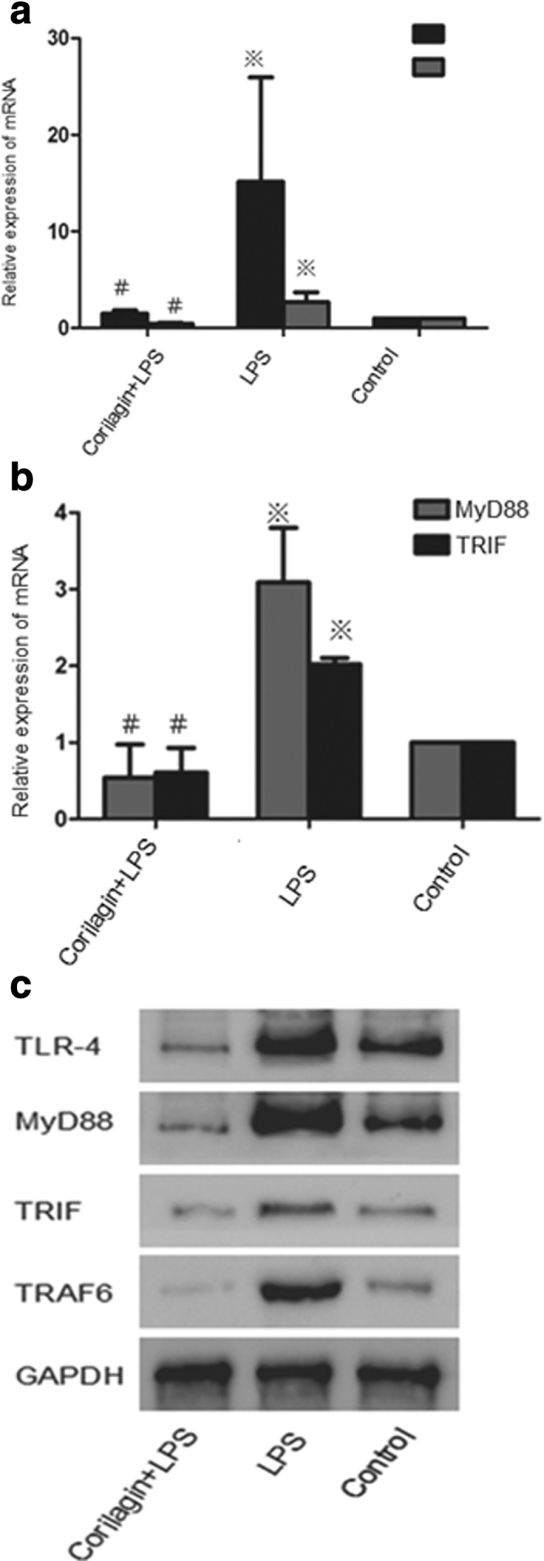
The mRNA and protein expression TLR4, TRAF6, MyD88 and TRIF in animal models. **a** Effect of Corilagin on mRNA expression of TLR4 and TRAF6 in animal model. **b** Effect of Corilagin on mRNA expression of MyD88 and TRIF in animal model. ※p < 0.01 compared with the control group. #p < 0.01 compared with the LPS group. **c** Effect of corilagin on TLR4, MyD88, TRIF and TRAF6 protein expression in animal model. Western blot assay showed the TLR4, MyD88, TRIF and TRAF6 proteins expression were greatly increased in LPS group, with Corilagin intervention, the proteins were decreased compared with that in the LPS group

In the LPS group the mRNA levels of TLR4, MyD88, TRIF and TRAF6 were significantly increased when compared with the control group (*p* < 0.01). With Corilagin intervention, the gene expression of TLR4, MyD88, TRIF and TRAF6 were significantly reduced when compared with the LPS group (*p* < 0.01) (Fig. 4a and b).

### Effect of Corilagin on protein expression of TLR4, MyD88, TRIF and TRAF6 in animal model

Western-blot assay was employed to test the changes of TLR4, MyD88, TRIF and TRAF6 proteins in the liver (Fig. 4C). The proteins were greatly increased in LPS group when compared with the control group, while in LPS + Corilagin groups the proteins were decreased when compared with the LPS group.

### Effect of Corilagin on expression of IL-1β, IL-6 in mice serum

**Fig. 5:**
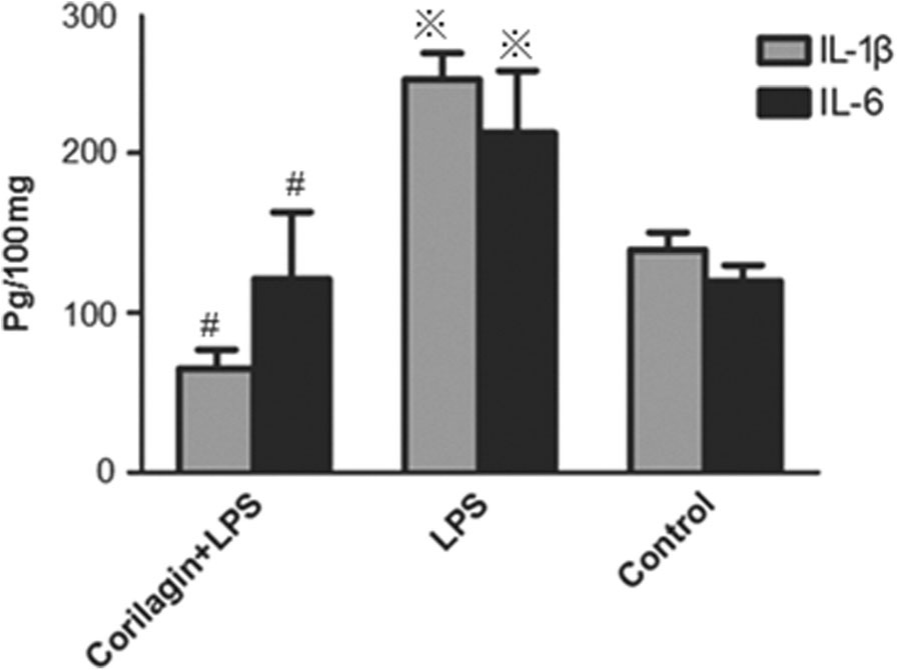
Effect of Corilagin on expression of IL-1β, IL-6 in mouse serum. Effect of Corilagin on expression of IL-1β, IL-6 in mouse serum. ※p < 0.01 compared with the control group. #p < 0.01 compared with the LPS group

The expression of IL-1β and IL-6 in mouse serum was tested by ELISA. In the LPS group the IL-1β, IL-6 levels were significantly increased when compared with the control group (*p* < 0.01). With Corilagin intervention, IL-1β, IL-6 expression were significantly reduced when compared with the LPS group (*p* < 0.01) (Fig. 5).

### Effect of Corilagin on liver histopathology

**Fig. 6:**
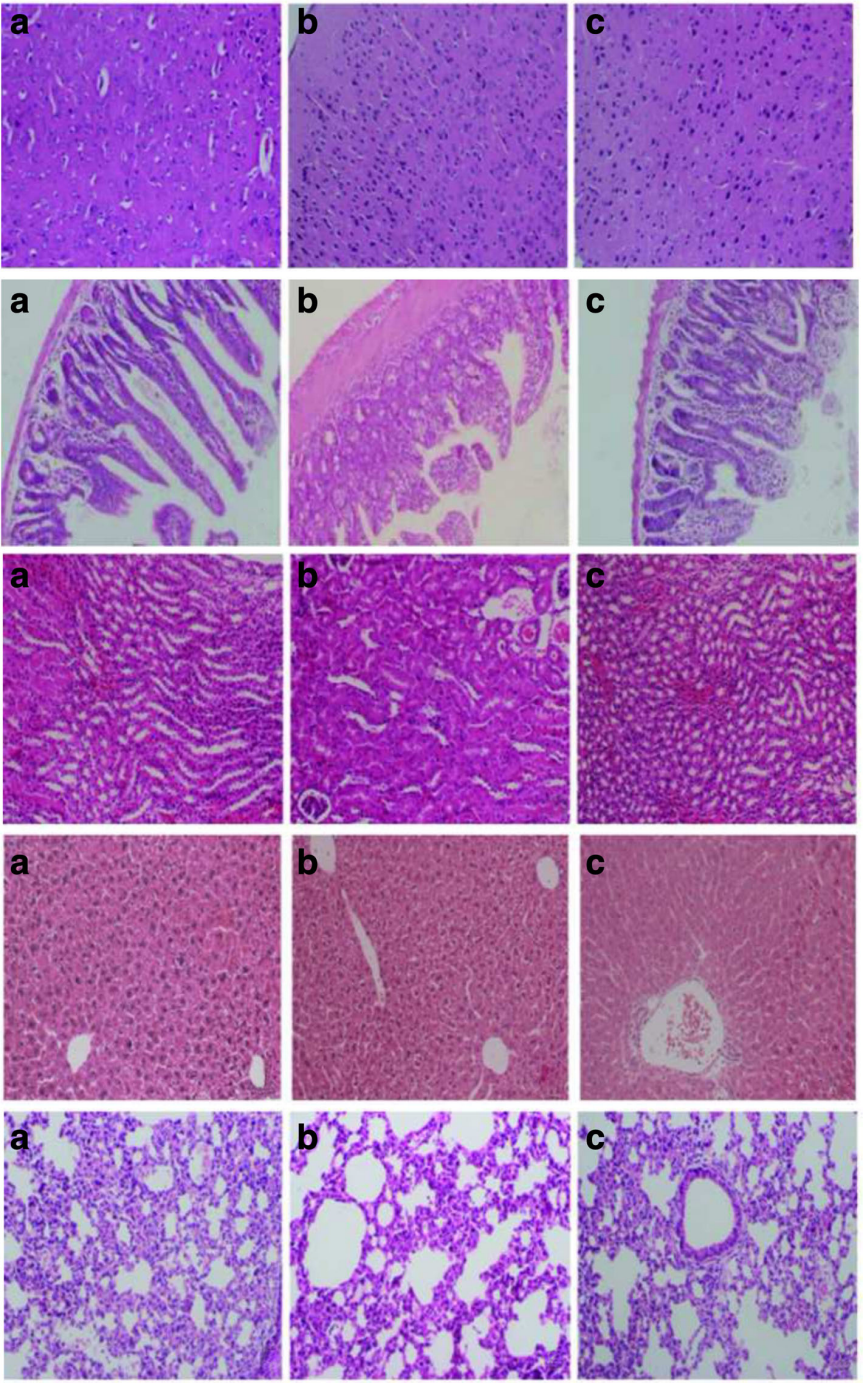
Pathological changes have been observed. **a** LPS + Corilagin group; **b** LPS group; **c** Control group. In the liver, pathological changes were not obvious. In lung and brain, interstitial cells showed obvious edema. There was no obvious changes in colon and kidney

Pathological changes have been observed in the liver, colon, lung, brain and kidney tissues. In the liver, pathological changes were not typical. In lung and brain, interstitial cells showed obvious edema. There were no obvious changes in colon and kidney (Fig. 6).

## Discussion

As important pattern recognition receptors (PPRs), TLRs play a pivotal role in innate immunity. TLRs identify a variety of pathogen-associated molecular patterns (PAMPs) or danger-associated molecular patterns (DAMPs), including teichoic acid, LPS, peptidoglycan etc. [27]. TLRs express on sorts of immune cells such as macrophages, master cells, monocytes, neutrophils, eosinophils, and so on. As an important molecule of acquired immune, TLR4 activates mononuclear phagocyte system, leading to a cascade of inflammation through MyD88 and TRIF pathways resulting in SIRS and sepsis eventually [28]. Therefore, regulating the TLR4 signaling pathways has become an important therapeutic target in sepsis [5].

Currently, medications for TLR4 were explored in labs and preclinical researches. TLR4 antagonist Eritoran have been used in experimental study and the efficacy and safety of Eritoran for clinical use are under further evaluation [29]. An anti-TLR4/MD2 monoclonal antibody could decrease lethality in the process of sepsis caused by implantation of a stent in mice [30]. Studies also have shown that blocking TLR2 or TLR4 resulted in decreasing disease severity in sepsis [31]. In our study, we evaluated the therapeutic potential of Corilagin in sepsis with hope to find a new way to control the extreme inflammatory status of sepsis.

MyD88 and TRIF pathways are the downstream molecules of TLR4. MyD88 pathway is the classical pathway, inducing production of pro-inflammatory cytokines. TRIF pathway produces pro-inflammatory cytokines and type I interferon simultaneously [32]. In our study, we looked forward to finding whether Corilagin contains the anti-inflammatory ability and which way it works. Therefore we selected key signaling molecules TLR-4, MyD88, TRAF6 and TRIF to evaluate the effect of Corilagin in TLR4 signaling pathway.

TLR4 signal can be beneficial in pathogen removing at early stage, but subsequent cascade release of pro-inflammatory cytokines may cause lethal effects [33]. In our study, with Corilagin intervention the expression of TLR4 were greatly decreased in the inflammatory phase, resulting in lowered expression of downstream molecules MyD88, TRAF6, TRIF and inflammatory cytokines IL-1β and IL-6.

TLR4 sharing with TRL2 downstream molecules TIRAP, and then the signal activated MyD88. As the important downstream molecular of MyD88, TRAF6 expression was reduced in the study. Furthermore, TLR4 sharing TRIF signaling pathway with TLR3, which can also activates TRAF6 in producing pro-inflammatory cytokines. Corilagin showed the capability in down-regulating TRAF6 and TRIF signaling pathway in extreme inflammation status, which demonstrated that Corialgin might have the potential to control outbreak of inflammatory cascade in sepsis.

IL-1β is mainly generated by monocytes. It promotes the production of NO, and then aggregates the inflammatory chemokines and adhesion molecules to expand the inflammatory response. IL-6 also is a monocyte-derived cytokine, as an important acute phase reaction medium in promoting inflammation [34]. In our study, Corilagin reduced the production both *in vitro* and *in vivo*, indicating Corilagin possessed the anti-inflammatory capability.

## Conclusion

In our study, Corilagin showed capability of inhibiting the expression of TLR4 signaling molecules in both MyD88 and TRIF signaling pathways, which demonstrated that Corilagin might be a potential agent to rescue the patients from extreme inflammatory status in sepsis.
